# Light-Induced Therapies for Prostate Cancer Treatment

**DOI:** 10.3389/fchem.2019.00719

**Published:** 2019-10-29

**Authors:** Claudia Ferroni, Alberto Del Rio, Cecilia Martini, Elisabetta Manoni, Greta Varchi

**Affiliations:** ^1^Institute of Organic Synthesis and Photoreactivity – ISOF, Italian National Research Council, Bologna, Italy; ^2^Innovamol Consulting Srl, Modena, Italy

**Keywords:** prostate cancer, light, photodynamic therapy, photothermal therapy, nanoparticles, targeted delivery

## Abstract

Prostate cancer (PC) is one of the most widespread tumors affecting the urinary system and the fifth-leading cause from cancer death in men worldwide. Despite PC mortality rates have been decreasing during the last years, most likely due to an intensification of early diagnosis, still more than 300,000 men die each year because of this disease. In this view, researchers in all countries are engaged in finding new ways to tackle PC, including the design and synthesis of novel molecular and macromolecular entities able to challenge different PC biological targets, while limiting the extent of unwanted side effects that significantly limit men's life quality. Among this field of research, photo-induced therapies, such as photodynamic and photothermal therapies (PDT and PTT), might represent an important advancement in PC treatment due to their extremely localized and controlled cytotoxic effect, as well as their low incidence of side effects and tumor resistance occurrence. Based on these considerations, this review aims to gather and discuss the last 5-years literature reports dealing with the synthesis and biological activity of molecular conjugates and nano-platforms for photo-induced therapies as co-adjuvant or combined therapeutic modalities for the treatment of localized PC.

## Introduction

Prostate cancer (PC) is one of the most common tumors in the urinary system and a serious threat to men's health. In the USA, the estimated new cases for year 2019 are 174,650 with a number of estimated deaths of 31,620, ranking just second among malignant tumors in men (Siegel et al., 2019). At diagnosis, the nearly 90% of PC cases are localized in the organ or only spread in the nearby tissues. In these cases, the decision on possible clinical treatments depends on different parameters, such as the tumor stage and levels of prostate-specific antigen (PSA) and it is usually in favor of active surveillance, prostatectomy or local radiotherapy (Lepor, 2004; Ouzzane et al., 2017). Once the disease has spread outside the prostate, androgen deprivation therapy (ADT) by surgical or chemical castration is preferred in order to decrease circulating testosterone levels. Unfortunately, the response to this therapy is transitory and most patients will develop resistance leading toward castration-resistant prostate cancer (CRPC) after 18–36 months (Nevedomskaya et al., 2018). At this stage, conventional treatments such as traditional chemotherapy or radiotherapy have numerous limitations, including poor patient compliance, bad tissue selectivity, severe toxicity, and drug resistance issues.

Due to the high incidence of PC, researchers all over the world have focused on developing new drugs and/or drugs combination for challenging this disease. Several clinical trials are ongoing comprising different approaches, such as (i) targeting the androgen receptor signaling; (ii) inhibiting the P13K7AKT/mTOR pathway; (iii) addressing the DNA damage repair pathway, and (iv) inhibiting specific epigenetic mechanisms (Nevedomskaya et al., 2018). However, to discuss recent advances in the clinical treatment of prostate cancer, including novel targeted chemotherapeutic approaches, and immunotherapy as well as progresses in molecular characterization of PC through omics techniques, has been extensively reviewed elsewhere (Nevedomskaya et al., 2018; Barata and Sartor, 2019; Dess et al., 2019) and falls beyond the scope of this review.

Despite these advances, there is still a need of novel and more effective clinical options able to tackle the fast-mutating nature of PC disease, while lowering the number of side effects that may arise from any kind of treatment.

Among the possible alternatives, researchers have pointed their attention to unconventional therapeutic modalities and/or a combination of them in order to achieve better clinical outcomes for PC patients. In the last four decades, light-induced therapies, such as photothermal (PTT) and photodynamic therapy (PDT), have been increasingly studied as alternative and/or co-adjuvant strategies for improving patients survival (Dos Santos et al., 2019). Indeed, in view of their high selectivity and minimal invasiveness, these near-infrared (NIR)-based phototherapeutic approaches, emerged as powerful techniques for PC treatment (Zhang et al., 2017).

Besides covering the basic working principles of PTT and PDT, this paper will review the last 5-years of literature reports on the use of PDT and PTT-mediated treatment of PC.

Major advantages of light-induced therapies as respect to classical chemo- and radio-therapeutic approaches are the following: (i) the intrinsic ability of some photoactive molecules to preferentially localize in the tumor, which in turn allows for reduced systemic toxicities; (ii) the possibility of inducing the formation of cytotoxic species only under the exclusive control of light at a specific wavelength; (iii) the low incidence of resistance phenomena; and (iv) the reduced number of side effects, thus favoring the overall patient compliance.

**Figure 1:**
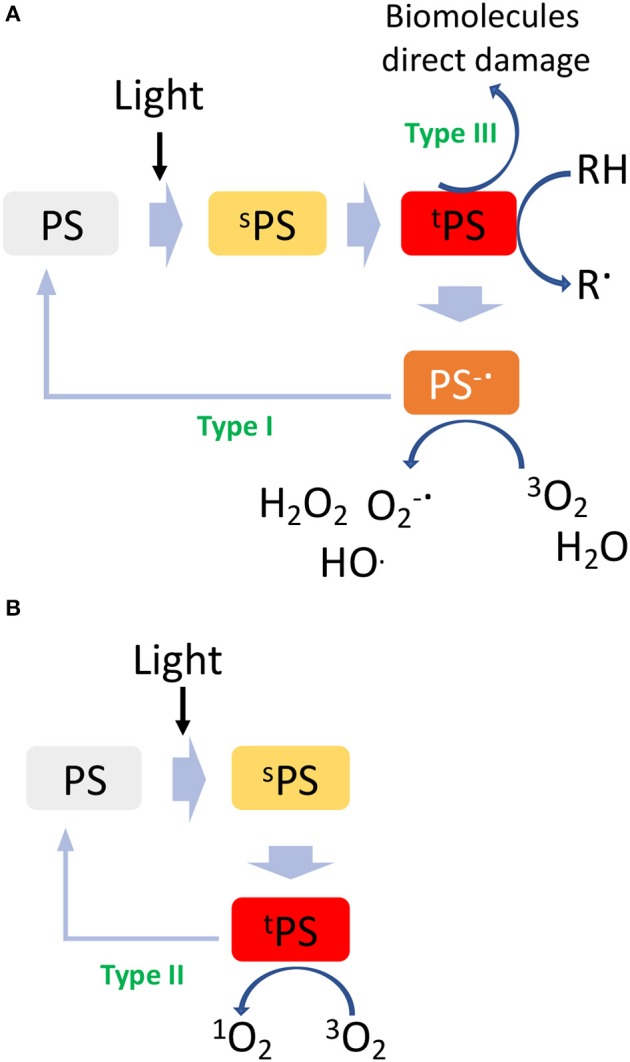
Graphical sketch of type I-III **(A)** and type II **(B)** PDT mechanism of action.

Photodynamic therapy involves biomolecules photooxidation through two distinct mechanisms, e.g., type I and type II. In particular, upon irradiation with light at a specific wavelength, a molecule called photosensitizer (PS) is activated to an excited singlet state (^s^PS, Figure 1A) by absorbing a photon. From this excited singlet state, the PS can translate into a triplet state (^t^PS, Figure 1A), where a hydrogen atom or an electron transfer can take place between the ^t^PS and the surrounding substrates leading to the formation of free radicals, which then interact with ambient triplet oxygen (^3^O_2_) and H_2_O to generate reactive oxygen species (ROS) (type I, oxygen-independent process, Figure 1A). On the other hand, the type II pathway involves an energy transfer directly between ^t^PS and ^3^O_2_, resulting in the formation of a highly reactive singlet oxygen (^1^O_2_, Figure 1B) (Mokwena et al., 2018). In both cases, the highly reactive radicals and oxygen species formed during this process react with biomolecules, i.e., DNA, lipoprotein, etc., inducing severe cells damage and death. A type III mechanism could also take place in case the excited ^t^PS reacts directly with surrounding biomolecules. By analyzing the PDT mechanism, it is evident that this technique gains therapeutic effectiveness only if all conditions are satisfied at the same time: the presence of a PS, of oxygen and/or oxidizable molecules and light; unless these are not achieved, PS alone is non-toxic for the patient. In this sense, while PDT is a highly selective approach, the conditions to be achieved are very demanding since deep tumors are difficult to be reached by light and most often, they lack of sufficient oxygen concentration in turn limiting the overall efficacy of the process (Lian et al., 2017).

**Figure 2:**
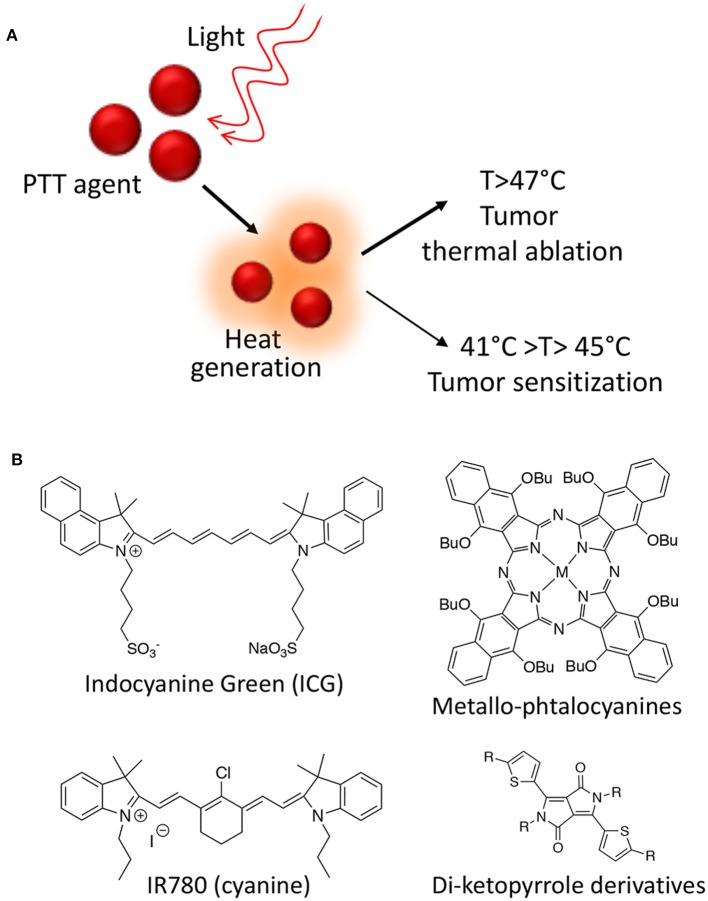
Photothermal therapy mechanism of action **(A)**; Class of organic molecules able to act as PPT agents **(B)**.

Besides ROS, heat is also a powerful mean of inducing cancer cells death. Hyperthermia for cancer therapy is defined as a treatment in which the target tissue is exposed to high temperatures that either destroy the tissues directly (thermal ablation with T > 47°C) or render the cancer cells more susceptible to other treatment modalities (thermal sensitization 41°C > T > 45°C) (Figure 2A).

Several photothermal therapy (PTT) agents are known from the literature including both organic molecules and nanoparticles (NPs). From a photo-physical point of view, an ideal PTT agent have essential prerequisites in order to be effective: it should have a strong absorbance in the NIR region, while displaying low fluorescence quantum yield and low ^1^O_2_ generation yield in order to maximize the conversion of absorbed photons into heat. Moreover, the PTT agent has to be non-toxic in the absence of light irradiation and to degrade or to be excreted quickly in order to improve safety (Deda and Araki, 2015). Among organic molecules able to act as PTT agents, indocyanine green derivatives, phthalocyanines, cyanines, and di-ketopyrrolopyrrole derivatives are the most effective and the most studied so far (Figure 2B) (Cai et al., 2017).

However, due to their intrinsic properties, nano-PTT agents have gathered the highest interest in recent years. Among those, metallic NPs, such as gold, palladium, and cupper, have shown the highest efficacy in the thermal ablation of cancer both *in vitro* and *in vivo*, alone or in combination with other therapies due to their unique plasmonic properties, which have been heavily reviewed in the literature (Doane and Burda, 2012; Jaque et al., 2014).

## Molecular Conjugates for PDT-mediated PC Treatment

Present strategies for cancer therapies often focus on both minimizing unwanted toxicity and enhancing therapeutic efficacy. This is often achieved by conjugating drugs with targeting agents or by combining two different drugs through self-immolative linkers in order to control their delivery at the target site (Tranoy-Opalinski et al., 2008). This approach applied to PDT and PTT agents is of particular interest since photo-active molecules are effective even when bound to other molecular scaffolds.

**Figure 3:**
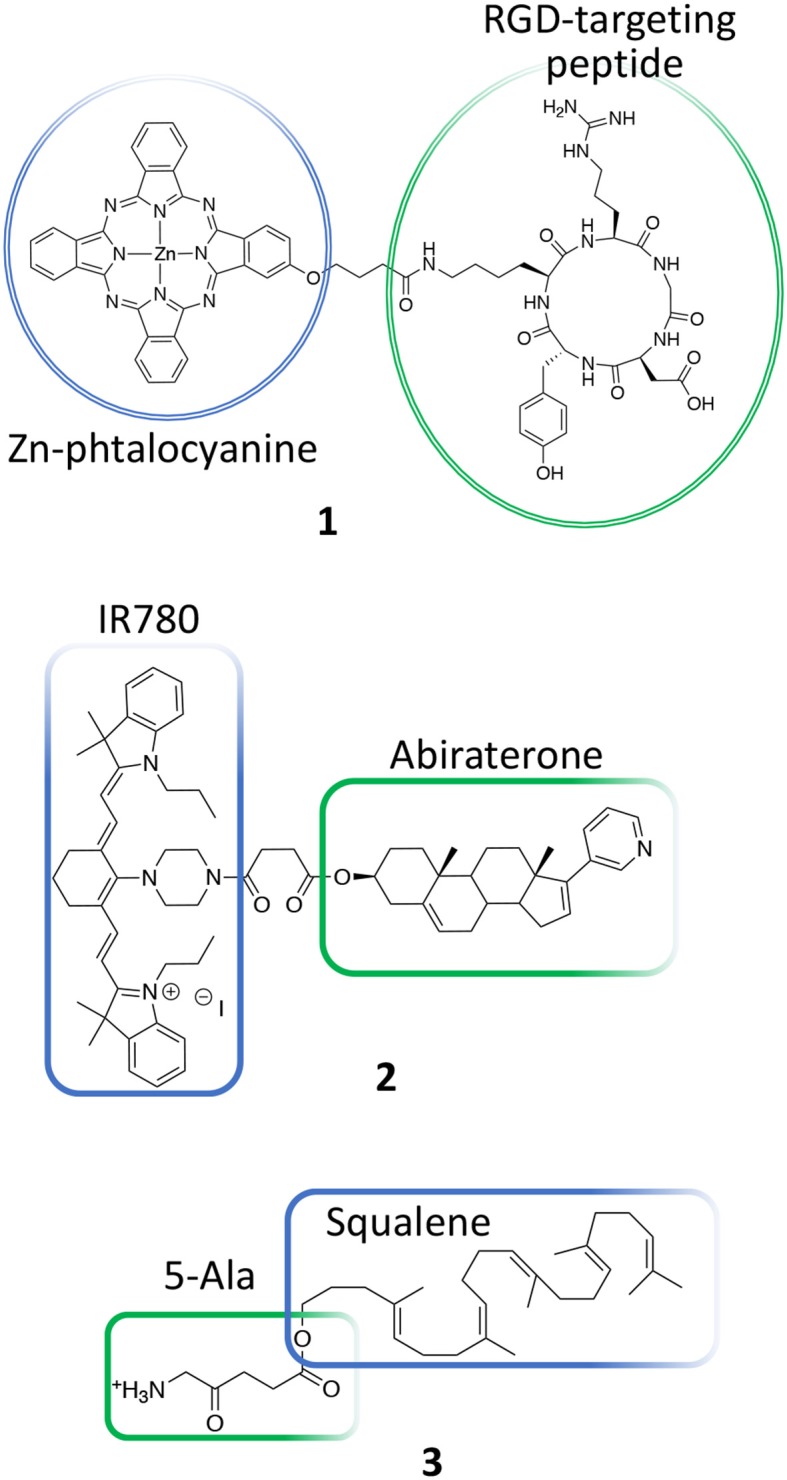
Chemical structures of conjugates **1**, **2**, and **3**.

Based on these considerations, several PDT conjugates have been recently reported for treating PC. Luan et al., for instance, reported the synthesis of a novel unsymmetrical phthalocyanine conjugated with a cyclic arginine-glycine-aspartic acid sequence (RGDyK) (**1**, Figure 3) (Luan et al., 2016). This pendant was specifically selected due to its higher proteolytic stability as compared to linear peptides and because it is known to be highly selective for α_ν_β_3_ integrin overexpressed on cancer cells and on tumor blood vessels (Desgrosellier and Cheresh, 2010). In details, authors report the *in vitro* PDT efficiency of their conjugate on DU145 prostate cancer cell line that are known to overexpress α_ν_β_3_ integrins as well as their preferential cellular uptake. Overall, their results demonstrate that upon conjugation with the RGD cyclic peptide, the Zn-phtalocyanine show similar photochemical properties, being able to induce highly comparable IC_50_ values in DU145 cells, e.g., 0.05 vs. 0.04 μM for free Zn-phtalocyanine and compound **1**, respectively. Interestingly, the RGD-modified sensitizer showed improved cellular uptake as respect to the untargeted sensitizer in DU145 cells (Table 1, entry 1).

**Table 1:** *In vtiro* and *in vivo* settings of different PDT and PTT mediated therapies of prostate cancer.

			***In vitro*** **settings**				***In vivo*** **settings**					
		**PS/drug**	**Cells lines**	**Dose**	**Irradiance–fluence**	**Light source/ wavelength**	**Animal model**	**Cells lines**	**Dose**	**Irradiance–fluence**	**Light source/ wavelength**	**References**
Molecular Conjugates—PDT	**1**	Phthalocyanine conjugated with a cyclic RGDyK sequence (Cmpd. **1**, Figure 3)	DU145	0.078; 0.16; 0.31; 0.63; 1.25; 2.5;10.5 μM	10 mW/cm^2^-12 J/cm^2^	Red LED array/660 nm	n.a.	n.a.	n.a.	n.a	n.a.	Luan et al., 2016
	**2**	IR780-abiraterone conjugate (ABI-780, Cmpd. **2**, Figure 3)^a^	PC3, DU-145, C4-2^b^ LNCaP and RWPe-1	2.5; 5; 10; 20; 40; 80; 160 μM Cells were tested only for selective uptake studies	n.a.	n.a.	Athymic nude mice; subcutaneous xenograft	LNCaP	IR-780 group (3.34 mg/kg/d); Abi group (3.5 mg/kg.d); Abi-780 group (5.75 mg/kg.d); i.p. injection	n.a^c^	n.a.	Yi et al., 2016
	**3**	PpIX-polyamines ponjugates (Cmpds. **5** and **6** **Figure 4**)	PC-3, DU-145, LNCaP and non-malignant RWPE-1	10–200 ng/mL (PpIX)	n.a.-−75 J/cm^2^	PDT TP-1 (Cosmedico Medizintechnik GmbH)/630 nm	5-weeks-old female BALB/c nu/nu mice; subcutaneous xenograft	PC3	10 mg/Kg	n.a.−200 J/cm^2^ (2 × 5 min every 5 min)	Led laser H-series/660 nm	Fidanzi-Dugas et al., 2017
	**4**	Pheo a/peptide/PSMA (Cmpd. **7**, **Figure 5**)	(PSMA+) PC3pip; (PSMA-) PC3flu; PC3-ML-1124; PC3- ML-1117^d^	n.a.	n.a.	n.a.	Athymic male nude mice; subcutaneous xenograft	PSMA+ PC3 PIP or PSMA- PC3 flu cells	30 and 50 nmol	55 mW/cm^2^-100 J/cm^2^	Laser/671 nm	Harmatys et al., 2018
	**5**	PSMA-targeted Pc413 and IR700 (Cmpds. **9** and **10** **Figure 7**)	(PSMA+) PC3pip; (PSMA-) PC3flu	1 μmol/L	8.3 mW/cm^2^-0.5 J/cm^2^	Apollo Horizon Projector/ >500 nm	6 to 8-weeks-old male athymic; subcutaneous xenograft	PSMA+ PC3 PIP	Cmpd. **9**: 0.1 mg/kg; 0.25; 0.5 mg/kg; irradiated once 24 h post-injection Cmpd. **10**: 0.25, 0.5 mg/kg on days 0, 4, and 8 and irradiated 1 h post- injection on the 3 days	Cmpd. **9:** 33.3 mW/cm2–150 J/cm2 Cmpd. **10:** 31.8 mW/cm^2^-50 J/cm^2^	Cmpd. **9:** laser diode equipped with fiber optic/672 nm Cmpd. **10:** diode LED light/690 nm	Wang X. et al., 2016
Nanoparticles mediated PDT	**6**	AlPcS_4_@PMMA NPs	PC3	18 μg/mL	876.6 mW/cm^2^-263 J/cm^2^ or 1,581 J/cm^2^	Red LED light/668 nm	Adult 6-weeks-old SCID mice; subcutaneous xenograft	Luciferase Expressing PC3 (PC3-luc)	Intratumor injection 25 μg/mL (2 treat./wks for 4 wks)	26.8 mW/cm^2^-8.04 J/cm^2^	Red LED light/668 nm	Duchi et al., 2016
	**7**	ClAlPc@NC ClAlPc@NE	LNCaP	0.3 μg/mL	n.a.−4 J/cm^2^ or 7 J/cm^2^	Diode eagle laser/670 nm	na	na	na	na	na	Leandro et al., 2017
	**8**	PSMA-1@NPsPc4	(PSMA+) PC3pip; (PSMA-) PC3flu	0.2 μmol of Pc4	n.a.−0.1; 0.5 and 1 J/cm^2^	Diode Laser/672 nm	6–8-weeks-old male athymic nude mice; subcutaneous xenograft	GFP-expressing PC3pip cells	0.07 mg/kg (with respect to Pc4) via tail vein	0.1 W/cm^2^-150 J/cm^2^ or 300 J/cm^2^	Diode Laser/672 nm	Mangadlao et al., 2018
	**9**	PGL@MBs (US and PDT combination)	PC3	0.2 μM-1 μM	300 mW/cm^2^-180 J/cm^2^	Xenon lamp with a filter passing light (650 nm) + low-frequency US	5–6-weeks-old male BALB/c athymic nude mice; subcutaneous xenograft	PC3	5 mg/kg intravenous	200 mW/cm^2^-360 J/cm^2^	Laser equipped with optical fiber/650 nm	You et al., 2018
	**10**	Fe_3_O_4_-Ce6-FA	PC3	6.25; 12.5; 25; 50; 100 μg/mL	20 mW/cm2–36 j/cm2	Red LED light/660 nm	n.a.	n.a.	n.a.	n.a	n.a.	Jung et al., 2018
	**11**	Fe_3_O_4_-Rose Bengal ROS responsive NPs	Tramp-C1	32 μM (Rose Bengal)	100 mW/cm^2^-30 J/cm^2^	Laser/532 nm	n.a.	n.a.	n.a.	n.a	n.a.	Yeh et al., 2018
Photo-thermal therapy	**12**	PDA-PAH-c Doxorubicin NPs	PC3, DU145, LNCaP	Range: 10-100 μg/ml (Dox)	2 W/cm^2^-1,800 J/cm^2^	Continuous-wave laser diode/808 nm	Male Balb/c mice; subcutaneous xenograft	PC3	n.a.	1 W/cm^2^-9000 J/cm^2^	Continuous-wave laser diode/808 nm	Zhang et al., 2017
	**13**	Silver gold nanoshell (SGNS) 5-Fluoroacil	PC3, DU145	Range: 0–16 μM (5-FU)	0.8 W/cm^2^-120 J/cm^2^	Continuous-wave laser diode/808 nm	n.a.	n.a.	n.a.	n.a	n.a.	Poudel et al., 2018
	**14**	TAT-gold nanostars/MSCs	PC3, DU145, LNCaP	0-160 pM of TAT-GNS	2.5 W/cm^2^-450 J/cm^2^	Continuous-wave laser diode/808 nm	Nude mice; subcutaneous xenograft;	PC3	**Intratumor** 43.73 μg **Intravenous** 218.65 μg	**Intratumor**: 1.0 W/cm^2^-600 J/cm^2^ **Intravenous**: 1.5 W/cm^2^-900 J/cm^2^	Continuous-wave laser diode/808 nm	Huang et al., 2019
	**15**	Anti-PSMAmab-IR700	(PSMA+) PC3pip-luc; (PSMA-) PC3flu	3 μg/mL	50 mW/cm^2^-range: 0–32 J/cm^2^	Red LED light/690 nm	6–8 weeks old female athymic nude mice; subcutaneous xenograft;	PC3pip-luc	100 μg, intravenous injection	50 mW/cm^2^-50 J/cm^2^ (day 1); 100 J/cm^2^ (day 2)	Laser/690 nm	Nagaya et al., 2017
Combined treatments	**16**	P13K pathway inhibitors (BYL719; BKM120; BEZ235) + Verteporfin	SV40 immortalized mouse endothelial cells; PC3^e^	P13K inhibitors (0–500 nmol/L) Verteporfin 200 or 400 ng/mL	5 mW/cm^2^-0.5 J/cm^2^	Diode laser/690 nm	6–8 weeks old male athymic nude mice; subcutaneous xenograft	PC3	**BEZ235**: 40 mg/kg/day for 24 days (oral gavage; 1 h before PDT treatment); **Verteporfin**: 0.5 mg/kg (intravenous injection; 15 min before irradiation)	50 mW/cm^2^-30 J/cm^2^	Diode laser/690 nm	Kraus et al., 2017
	**17**	Cisplatin prodrug [Pt(IV)] + melanin nanoparticles (PTT)	PC3; DU145; LNCaP	5 μM of Platinum	0.5 W/cm^2^-450 J/cm^2^	Continuous-wave diode laser/808 nm	Male Balb/c nude mice; subcutaneous xenograft	PC3	10 μmol Pt/kg on day 1	0.3 W/cm^2^-180 J/cm^2^	Continuous-wave diode laser/808 nm	Zhang et al., 2018
	**18**	ICG-Ce6 HSA NPs	PC3	**Ce6**:1.25 μg/ml; **ICG**: 5 μg/ml	0.2 W/cm^2^-72 J/cm^2^ 0.2 W/cm^2^-72 J/cm^2^ (660 nm) + 1 W/cm^2^-300 J/cm^2^ (808 nm) **iii)** 1 W/cm^2^-300 J/cm^2^ (808 nm) + 0.2 W/cm^2^-72 J/cm^2^ (660 nm)	Laser/660 nm + 808 nm	Nude mice; subcutaneous xenograft	PC3	**Ce6** 2.5 mg/Kg	0.2 W/cm^2^-144 J/cm^2^ 0.2 W/cm^2^-144 J/cm^2^ (660 nm) + 1 W/cm^2^-300 J/cm^2^ (808 nm) **iii)** 1 W/cm^2^-300 J/cm^2^ (808 nm) + 0.2 W/cm^2^-144 J/cm^2^ (660 nm)	Laser/660 nm + 808 nm	Ji et al., 2019

a IR780 is used only for cancer cells targeting and in vivo NIRF live imaging purposes;

b C4-2 are androgen independent, metastatic subline derived from LNCaP cells;

c Efficacy is due to Abiraterone only; IR780 favors tumor localization;

d In vitro studies focused on cellular uptake only;

e *Cells were incubated with P13K inhibitor for 1 h prior to PDT treatment*.

Another recent example of molecular conjugate for treating PC combines IR-780 dye and abiraterone. IR-780 iodide is a lipophilic cyanine with an emission peak at 780 nm that has been used for cancer imaging and therapy including PDT and PTT (Wang K. et al., 2016; Tran et al., 2017). In particular, this dye is known to selectively accumulate in cancer cells and has been used to discriminate between tumor lesions from normal tissues (Zhang et al., 2014). Yi et al. recently described on a novel IR780-abiraterone conjugate (**2**, Figure 3) for the concomitant imaging and therapy of PC both *in vitro* and *in vivo* (Yi et al., 2016).

Abiraterone is a CYP17 inhibitor and acts as an antagonist of the androgen receptor through the inhibition of the 3β-hydroxysteroid dehydrogenase, which is involved in dihydrotestosterone synthesis in castration-resistant PC (CRPC) (Yin and Hu, 2014). Unfortunately, the daily use of abiraterone is often associated with toxicity; thus, authors propose the chemical conjugation between abiraterone and IR-780 (**2**, Figure 3) in order (i) to minimize abiraterone side effects by exploiting the IR780 preferential accumulation in the tumor tissue and (ii) to combine abiraterone therapeutic effect with the fluorescence imaging properties of this novel conjugate for tumor imaging. The presented data show that the new compound maintained the preferential accumulation of IR-780 in cancer cells and exerted a synergized tumoricidal activity against PC cells in comparison with IR-780 or abiraterone alone. In particular, the abiraterone-IR780 conjugate showed a dose-dependent inhibition of cells proliferation on both LNCaP and C4-2 cells (IC_50_ 4.17 and 8.36 μM, respectively), that are representative models of androgen dependent and independent cell lines, respectively. Moreover, the conjugate significantly increased the percentage of apoptotic cells (LNCaP and C4-2 cells) and reduced of about 2-fold the migration and invasion potential of both LNCaP and C4-2 cells as compared to control groups. *In vivo*, the conjugate at the dose of 5.75 mg/kg/day was safe, and showed a more pronounced tumor inhibition effect as respect to IR780 and abiraterone alone, reaching an almost complete response 4 weeks after treatment than other treatments (Table 1, entry 2).

**Figure 4:**
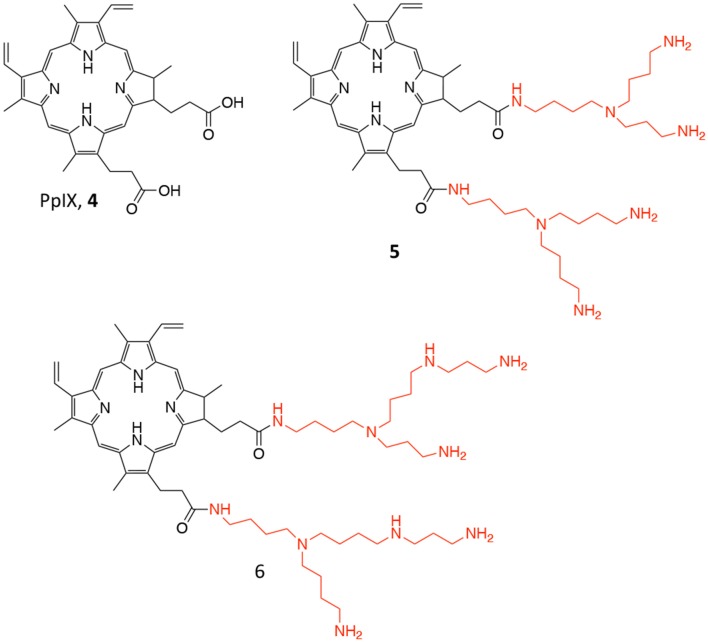
Chemical structure of the photosensitizer PpIX (**4**), and of its polyamine conjugates **5** (spermidine), and **6** (spermine).

Another PDT conjugate for PC treatment, was recently described by Lange's research group, which proposed the chemical combination of 5-aminolevulinic acid (5-ALA) and squalene in the form of nano-assemblies as potential candidate for the PDT treatment of PC (**3**, Figure 3) (Babič et al., 2018). Squalene is a precursor of cholesterol and other steroids; it is a natural compound synthesized in the human body by liver and it has found application in pharmacology as an excipient for the preparation of emulsions for the delivery of several active ingredients (Reddy and Couvreur, 2009). Specifically, this technological approach is named “squalenoylation” and comprises the formation colloidal nano-assemblies upon conjugation of the active drug with squalene (Couvreur, 2016). On the other side, 5-ALA is a small molecule that is metabolically converted in human body to protoporphyrin IX (PpIX, **4**, Figure 4), thus providing an efficient and non-toxic reservoir of photosensitizer for photo-therapy and photo-diagnosis (Fukuhara et al., 2013). In their work, authors describe the synthesis of the 5-ALA/squalene conjugate and its self-assembly in water to form 5-ALA/squalene nanoassemblies. Indeed, the paper only focuses on the conjugate synthesis and characterization, demonstrating that it is very efficient in producing PpIX in PC3 prostate cancer cells outperforming free 5-ALA in terms of fluorescence production, thus paving the way for a new and safe 5-ALA formulation (Babič et al., 2018).

Another approach involving PpIX was proposed by Fidanzi-Dugas and coworkers; in particular, the authors describe the conjugation of PpIX (**4**, Figure 4), with targeting molecules, i.e., polyamines, for achieving better PC selectivity and higher PDT efficacy (Fidanzi-Dugas et al., 2017). Polyamines, such as spermidine and spermine, are polycations endogenously produced by almost all cells and their production is especially high in rapidly growing cells due to the upregulation of the Polyamine Transport System (PTS) that occurs in these types of cells (Palmer and Wallace, 2010). In particular, the authors describe the conjugation of PpIX with two molecules of spermidine or spermine (**5** and **6**, respectively, Figure 4) through a flexible arm.

The ability of these derivatives to induce apoptosis in different PC cells lines was reported, as well as their *in vivo* efficacy in PC3 cells xenografts in nude mice. Interestingly, the reported data demonstrate that compounds **5** and **6** are more effective PS as compared to free PpIX in all tested cells lines; moreover, the study on apoptosis induction revealed that the PpIX-polyamine conjugates can inhibit anti-apoptotic pathways, by affecting Bcl-2, Akt, and NF-κB in human PC cells. Additionally, *in vivo* experiments showed that only compound **5** was effective in reducing tumor growth as respect to both compound **6** and freely administered ALA (Table 1, entry 3). Despite the authors report that ALA alone had only negligible effect on reducing tumor growth *in vivo*, control group did not comprise the administration of free PpIX, which would have been an interesting data to be compared.

**Figure 5:**
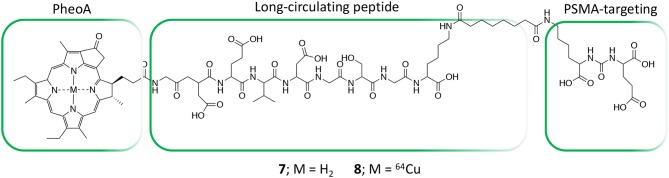
Structure of tri-modal conjugates **7** and **8** composed by Pheophorbide A, a long-circulating peptide and a PSMA targeting ligand.

In the field of conjugates compounds for the PDT treatment of PC, Zheng and coworkers recently reported the synthesis of a tri-modal conjugate combining a targeting moiety toward the prostate specific membrane antigen (PSMA), the photosensitizer pyropheophorbide a (PheoA) and a long-circulating peptide (**7**, Figure 5) (Chen et al., 2017; Harmatys et al., 2018). PSMA is a type II transmembrane glycoprotein that is highly overexpressed in PC and its expression has been proved to positively correlate with cancer aggressiveness (Flores et al., 2017).

**Figure 6:**
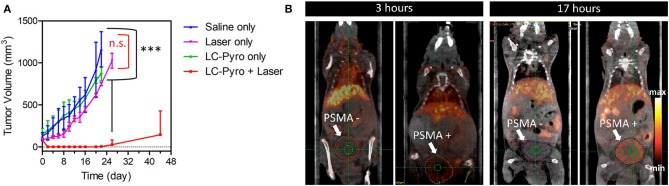
PDT efficacy of compound **7** PSMA-positive PC3pip subcutaneous tumor-bearing mice (*n* = 4 for each group, ***P ≤ 0.001, n.s., not significant) **(A)**. Enabled PET imaging enabled by compound **8** in an orthotopic prostate cancer model **(B)**. Reproduced with permission from Harmatys et al. (2018). Copyright (2018) American Chemical Society.

Indeed, the authors were able to demonstrate that the presence of a highly selective targeting PSMA moiety (Haberkorn et al., 2016) along with a peptide-based pharmacokinetic modulator was able to extend the conjugate plasma circulation time up to 10 h, resulting in a remarkably high tumor accumulation. In summary, the favorable pharmacokinetic of compound **7** as well as its ability to selectively bind PSMA, led to the effective single-dose tumor ablation by PDT in a PSMA-positive PC3pip subcutaneous mouse model (Figure 6A). Advantageously, the possible coordination of PheoA with radio-labeled ^64^Cu (**8**, Figure 5) also enabled PET imaging, confirming the selective accumulation of the conjugate in PSMA-positive PC3pip bearing mice as respect to PSMA-negative PC3flu inoculated mice (Figure 6B), that would possibly enable the planning of precise treatment schedules (Table 1, entry 4).

One major issue during the surgical removal of PC remains the identification and elimination of all cancerous tissues and cells without affecting surrounding tissues and nerves. In this view, the use of NIR fluorescence molecules able to act also as photosensitizers and kill residual cancer cells upon irradiation would be greatly beneficial.

**Figure 7:**
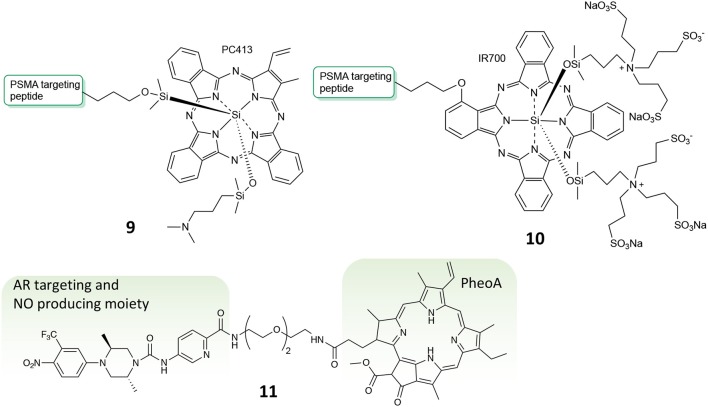
Chemical structures of compounds **9**, **10**, and **11**.

To find a molecule able to exert such features is the objective of the work described by Basilion and coworkers (Wang X. et al., 2016). In fact, their study aims at developing a PSMA-targeted PDT agent that could be used for surgical guidance and allow for subsequent PDT treatment to eradicate all residual cancer cells. In particular, the authors describe the synthesis, *in vitro* and *in vivo* biological activity of two different PMSA-PS conjugates, namely compounds **9** and **10** (Figure 7), and compared it with the un-conjugated PS. Their results demonstrate that the two PSMA-PS conjugates are selectively and specifically uptake by PSMA-positive PC3pip cells but not by PSMA-negative PC3flu cells. *In vivo* imaging studies showed that both **9** and **10** selectively accumulate in PSMA-positive PC3pip tumor mass, although **9** had better selectivity than **10**. Moreover, consistently with the uptake studies, *in vitro* phototoxicity showed that compound **9** was more potent against PSMA-positive PC3pip cells than for PSMA-negative PC3flu cells. This trend was confirmed also by *in vivo* experiments were one single dose of **9** was enough to considerably reduce tumor mass, in contrast to **10** that required 3-fold dose to achieve the same extent of tumor reduction (Table 1, entry 5).

Prostate cancer metastasis is a major issue in tumor management; metastases occur when primary cancer cells, named circulating tumor cells (CTCs), escape through the lymphatic and circulatory systems spreading to distal organs (Di Raimo et al., 2018; Pantel et al., 2019). In this view, Gaitas et al. proposed the removal of CTCs by means of an extracorporeal system based on the treatment of blood through a PDT approach. In detail, the authors report the synthesis of chlorin e6 conjugated to CD44 antibody (Ce6-CD44) able to selectively bind to PC tumor cells, for extracorporeal PDT performed *in vitro* both in well-plate ad in a tube (Kim and Gaitas, 2015). The authors demonstrated that, despite the settings difficulties, extracorporeal-PDT was scarcely effective in well-plate, due to the high light-absorbance by red blood cells, while in the tubing settings an almost complete CTCs reduction, e.g., PC3 cells, was achieved within 1 h upon 2 min of irradiation with a led array at 660 nm.

Another example of multi-modal conjugate for PC treatment was lately reported by our group, bearing two different functionalities, e.g., the PS PheoA and a molecular scaffold known for its ability to selectively bind the androgen receptor (AR) and to produce radical nitric oxide upon blue light illumination (**11**, Figure 7) (Rapozzi et al., 2015). Interestingly, our results confirmed that conjugate **11** was selectively uptake by PC cells that over-express the AR, e.g., VCap cells, as respect to those known as not expressing the receptor, i.e., PC3 cancer cells, thus demonstrating the effective *in vitro* selectivity of the reported system. Moreover, *in vitro* cytotoxicity studies revealed that compound **11** exerted much higher photo-toxicity when irradiated with white light as respect to red light only, thus demonstrating that besides ROS and ^1^O_2_ produced by PheoA irradiation, also nitric oxide is released when using a full visible spectrum light source (Rapozzi et al., 2017).

## Nanoparticles-Mediated PDT of Prostate Cancer

Although the great promise generated by PDT for cancer management, it still suffers from significant limitations which are in some cases due to the inherent properties of small molecules PS. In particular, most PS are poorly water soluble and can easily aggregate in this medium because of their hydrophobic and π-π stacking interactions, thus making it very difficult to formulate and possibly lowering their photodynamic efficiency, due to photobleaching and photoquenching phenomena (James et al., 2018). In addition, conventional PS show poor selectivity toward diseased tissues as compared to healthy ones and generally suffer from poor delivery to the target site. Also for these reasons, to date, the clinical translation of PDT has been hampered and currently PDT is mainly focused on the treatment of superficial cancers (Senior, 2005; Papakonstantinou et al., 2018). In the last decades, nanomaterials have been largely studied for both cancer therapy and diagnosis by exploiting the intrinsic characteristic of NPs based systems as compared to free drugs and/or diagnostic agents. Therefore, NPs have been extensively exploited also for delivery of PS taking advantage of the high surface-to-volume ratio that allows for high PS loading and subsequent reaching the effective concentration at the target site (Duchi et al., 2016). Moreover, the preferential PS delivery at the diseased tissue is achieved either by attaching tumor targeting ligands (active targeting) or through the enhanced permeation and retention (EPR) effect (passive targeting) (Maeda et al., 2000). Additionally, the ability to locate the PS within the NPs structure allows to protect it from possible photobleaching phenomena, therefore resulting in large extinction coefficients, higher quantum yields as well as improved water solubility (Lucky et al., 2015). Importantly, NPs might be simultaneously functionalized with diagnostic agents and/or loaded with multiple drugs, overall providing a multi-faced system for theranostic applications (Deda and Araki, 2015; Sivasubramanian et al., 2019).

In 2017 Leandro et al. reported the non-covalent entrapment of chloro-aluminum phthalocyanine (ClAlPc) within both nanocapsules (ClAlPc@NC) and nanoemulsions (ClAlPc@NE) and studied their effectiveness in the PDT treatment of PC (Leandro et al., 2017). Among the PSs, aluminum phthalocyanines are particularly promising due to their strong energy absorption and high fluorescence quantum yield; however, they are highly hydrophobic molecules and tend to aggregate, significantly reducing their efficacy. The work of Leandro compares the effect of ClAlPc encapsulation both in nanoemulsions and nanocapsules only *in vitro* against LNCaP cell lines (Table 1, entry 6). For clarity, nanoemulsions are fine oil-in-water dispersions were the lipophilic phase is constituted by oil, hydrophobic surfactants and water-miscible solvents, while the aqueous phase comprises water and hydrophilic surfactants. On the other hand, nanocapsules are sub-micron vesicular systems composed of an oily core surrounded by a thin polymeric membrane in which the hydrophobic drug can be located both in the oily core and into the polymeric membrane.

Through an in-depth confocal imaging study, the authors were able to observe that ClAlPc@NC were more efficiently internalized by LNCaP cancer cells as respect to nanoemulsions. These data were also confirmed by the photo-toxicity studies that account for higher efficacy of nanocapsules as respect to nanoemulsions especially at low light doses, e.g., 0.5 and 1.0 J/cm^2^.

**Figure 8:**
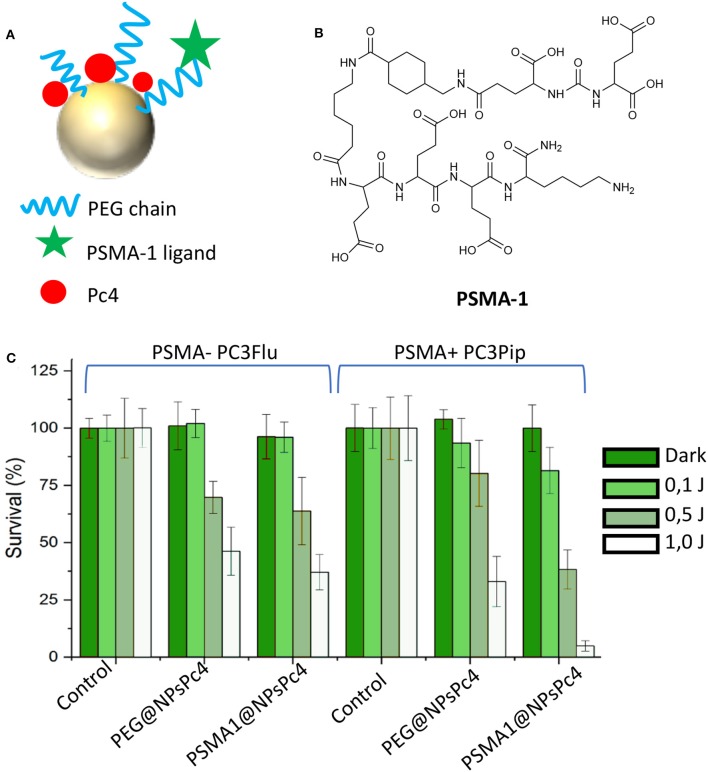
Representation of PSMA-targeted gold NPs non-covalently loaded with the phthalocyanine Pc4 **(A)**. Chemical structure of phthalocyanine Pc4 **(B)**. Efficacy of PSMA1@NPsPc4 in killing prostate cancer cell lines under different light dose conditions **(C)**. Panel **C** was adapted with permission from Mangadlao et al. (2018). Copyright (2018) American Chemical Society.

Another nanoparticles-based platform for the PDT treatment of PC was recently reported by Burda et al. (Mangadlao et al., 2018). In their paper the authors report the synthesis, characterization and *in vitro* and *in vivo* application of PSMA-targeted gold NPs non-covalently loaded with a phthalocyanine-based photosensitizer (Pc4) as a theranostic system for PC treatment (Figure 8A). The PSMA-1 ligand (Figure 8B), which was developed by the same research group in 2014 (Wang et al., 2014) was exploited as targeting antenna for PC tissues. Upon conjugation with a PEG chain, the linker was used for decorating gold NPs, while by exploiting the presence of the PEG corona, Pc4 was loaded onto NPs with no need of chemical functionalization.

Stable NPs were obtained with an average dry diameter of 20 nm and a loading ratio of about 20 Pc4 molecules *per* NPs, e.g., PSMA-1@NPsPc4. The affinity of the PSMA-targeted NPs was evaluated by competition binding assay in PSMA-positive PC3pip cells showing the lowest IC_50_ value for PSMA-1@AuNPs as compared to control, probably due to presence of multiple PSMA-1 ligands on the same NP. Interestingly, *in vitro* phototoxicity studies confirmed that PSMA-1@NPsPc4 were the most efficient and selective in killing PSMA-expressing PC3pip cancer cells as respect to PSMA-negative PC3flu cells and as respect to untargeted Pc4 loaded NPs (Figure 8C). *In vivo* experiments on PC3pip xenografts demonstrated that this nanosystems allows the visualization of the tumor enabling its resection upon light irradiation. Indeed, treated animals showed complete tumor remission at 14 days after treatment, demonstrating that PSMA-1 functionalized gold NPs are effective for both surgical guidance and for prostate tumor resection (Table 1, entry 8).

**Figure 9:**
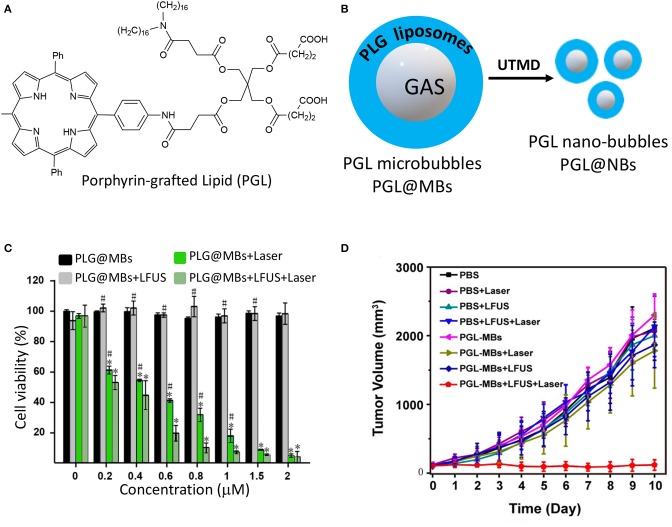
Chemical structure of porphyrin-grafted lipid (PGL) **(A)**; schematic representation of PGL liposomes surrounding microbubbles and their transformation into nanobubbles through UTMD **(B)**; PC3 cells viability under different treatments with increasing concentration of PGL using CCK-8 assay **(C)**; tumor growth-curves calculated by caliper measurements every day after treatments (*P < 0.05 vs. PGL-MBs only, ^#^*P* < 0.05 vs. PGL-MBs+LFUS+Laser). (Panels **C** and **D** were adapted and reprinted with permission from You et al., 2018).

An interesting application of nanotechnology for the PDT-guided treatment of PC involves the use of microbubbles. Microbubbles (MBs) are gas-filled structures stabilized by an external lipid layer, that are used as contrast agents for ultrasound (US) mediated clinical diagnosis (Ma et al., 2015; Dimcevski et al., 2016). In addition, they might represent promising platforms for cancer treatment by virtue of their responsiveness to US irradiation which is called Ultrasound Targeted Microbubble Destruction (UTMD), that enables the drug release through the cavitation effect (Lentacker et al., 2014). Major drawback of using microbubbles as delivery vehicles are i) their very poor loading ability of hydrophobic drugs, ii) their large sizes which often confine them in the blood vessels impeding their extravasation into the tumor mass. Very recently, Zheng and coworkers proposed a novel approach for obtaining porphyrin-loaded microbubbles, that are *in situ* transformed into NPs through UTMD (Table 1, entry 9) (You et al., 2018). In particular, the authors describe the synthesis of a porphyrin-grafted lipid (PGL) (Figure 9A), which self-assembles into NPs. They exploited the thin-film rehydration-sonication procedure in the presence of PGL for the synthesis of monolayer MBs in which the external shell is uniformly formed by liposomal PGL, e.g., PGL@MBs (Figure 9B). The selective accumulation of PGL@MBs within the tumor was then accomplished through UTMD technique which allowed the simultaneous transformation of MBs into nanobubbles (Figure 9B). PGL@MBs were obtained with a porphyrin loading content of 18.9 % w/w and when exposed to low-frequency US (LFUS) their hydrodynamic diameter went from 1 mm to nearly 100 nm without losing their ability to fluoresce and produce singlet oxygen. *In vitro* phototoxicity studies performed on PC3 prostate cancer cells, demonstrated that the combination of PGL@MBs + LFUS + laser increases cell death even at low concentrations, most probably due to the enhanced uptake of PGL as compared to the group without LFUS treatment (Figure 9C). Furthermore, *in vivo* experiments clearly showed a higher tumor localization of PGL@MBs after LFUS treatment as respect to control groups; similarly, the *in vivo* phototoxicity results confirmed the superior therapeutic efficacy of PGL@MBs plus US and irradiation (Figure 9D), thus representing a promising mean for translating microbubbles-based PDT systems to the clinical settings.

Magnetic NPs (Fe_3_O_4_) covalently functionalized with the PS chlorin e6 (Ce6) and folic acid as targeting agent were recently evaluated *in vitro* against PC3 cells by Jung and coworkers (Jung et al., 2018). In particular, the subsequent surface modification of Fe_3_O_4_ NPs with Ce6 and folic acid afforded Fe_3_O_4_-Ce6-FA NPs with uniform size around 20 nm. Particles displayed no toxicity in dark while they were very effective upon light irradiation in killing PC3 cells at a concentration of 6.25 μg/mL. In principle, this system represents an interesting theranostic candidate for *in vivo* experiments which should include MRI particles analysis, safety profile and efficacy on properly designed tumor model (Table 1, entry 10).

Cancer cells constitutively activate a number of growth factor pathways that are crucial for recruit nutrients, escape stress and proliferate. Moreover, their hyper-metabolism promotes the over-production of ROS from mitochondria and endoplasmatic reticulum, which has been correlated to genomic instability and tumorigenesis (Schieber and Chandel, 2014). In turn, ROS have been exploited as intracellular stimulus for selective anticancer drug releasing once in the presence of ROS-responsive materials (Yue et al., 2016). However, an efficient triggered ROS-mediated delivery remains a major challenge, especially for the minimal amount of ROS produced endogenously even in cancer cells. Based on these considerations, Huang and coworkers, recently proposed a Rose Bengal (RB) loaded core-shell NPs system constituted by a magnetic Fe_3_O_4_ core and an external shell of ROS responsive *branched-*PEI (*b*PEI), PVA and chitosan polymers, indicated by the authors as magnetic nanoclusters (MNCs) (Yeh et al., 2018). During their preparation, the MNCs were electrostatically loaded with negatively charged RB, which serves as photo-sensitive antenna for *b*PEI photo-oxidation, responsible for the release of drugs through the formation of ROS. In addition, the particles were loaded with paclitaxel for the combined photo and chemo-treatment of different cells lines *in vitro*, including Tramp-C1 prostate cancer cells and demonstrated the superior therapeutic efficacy of MNCs co-loaded with RB and the chemotherapeutic drug (Table 1, entry 11) (Chang et al., 2018).

## Photothermal Therapy of Prostate Cancer

Photothermal therapy (PTT) is a quite recently developed therapeutic strategy, which employs a combination of NIR laser light and photo-absorbing systems either to generate heat for thermal ablation of cancer cells or to induce the release of drugs (Zou et al., 2016). As for PDT, PTT exhibits unique advantages in cancer therapy including high specificity, minimal invasiveness and precise spatial-temporal selectivity (Hu et al., 2014). The therapeutic efficacy of PTT depends on the efficient transformation of light to heat by selected photothermal agents, which in turn should have strong NIR absorption within the biological transparent window, e.g., 700–1870 nm, in order to ensure the greater penetration depth into the tumor mass (Hemmer et al., 2017). Over the past decade, many different types of NIR-absorbing agents have been developed, such as metal-based and other inorganic nanomaterials and organic nanomaterials, thus providing a large spectrum of opportunities for this technique to access clinical practice (Phan et al., 2018). In this section, a selection of papers was reported on the PTT and or PTT-combined treatment of PC (Lee et al., 2017; Thapa et al., 2018).

**Figure 10:**
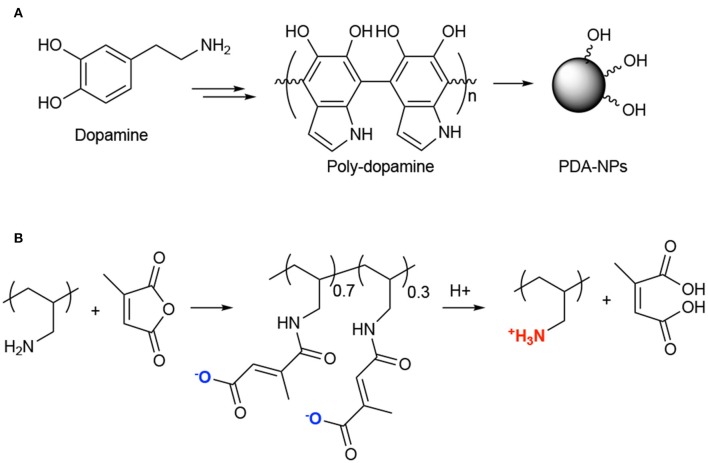
Synthesis of poly-dopamine NPs from dopamine **(A)**; Chemical structure of poly(allylamine)-citraconic anhydride (PAH-cit) and pH-dependent behavior **(B)**.

For instance, an application of PTT with chemotherapeutics was described couple of years ago by Guo and coworkers (Zhang et al., 2017). In particular, the authors proposed the synthesis and *in vitro* and *in vivo* application of multi-modal NPs composed of polydopamine (PDA) NPs (Figure 10A), poly(allylamine)-citraconic anhydride (PAH-cit) NPs and doxorubicin (Dox). Among the photothermal responsive materials, polydopamine is very fascinating because it has excellent biocompatibility, low cytotoxicity, it is unexpansive, very easy to functionalize and it has 40% of photothermal conversion efficiency upon NIR light absorption (Liu et al., 2013). PAH-cit NPs were selected by the authors for combination with polydopamine NPs due to their ability to switch from negatively to positively charged under acidic conditions, thus allowing the release of positively charged drugs through electrostatic repulsion (Figure 10B) (Chen et al., 2016). Combined NPs, namely Dox@PAHcit/PDA, of around 80 nm and a Dox loading of ca 30% were obtained; the release studies demonstrated that only under acidic conditions (pH = 5.5) Dox was released in a sustained manner up to 80% in 50 h. In addition, laser irradiation also favored the Dox release that was about 16% after 40 min irradiation as compared to 6% in absence of laser irradiation. *In vitro* results performed on PC3, DU145, and LNCaP cells, demonstrated similar activity in the dark (Dox activity), while the PTT effect was more evident in PC3 and DU145 as respect to LNCaP cell lines. Interestingly, *in vivo* experiments, performed in nude mice bearing PC3 tumors, showed that the Dox@PAHcit/PDA + laser irradiation provided the best therapeutic effect with no tumor recurrence 25 days after treatment (Table 1, entry 12).

Among PTT agents, gold NPs are probably the most suited and used due to their specific features (Stern et al., 2016; Riley and Day, 2017). Indeed, gold NPs exhibit a unique photo-physical property named localized surface plasmon resonance (LSPR) that is generated by the confined oscillation of free electrons cloud to their lattice upon irradiation with light of resonant frequency. Depending on its size, this resonance relaxes by non-radiative process in the form of heat. Despite their potential as PTT agents for cancer treatment, gold NPs suffer of several drawbacks, such high aggregation phenomena. To tackle these issues, Yong and coworkers reported a novel system constituted by silver-gold nanoshells (SGNS) incorporated within mesoporous silica (MS) shell loaded with 5-fluorouracil (5-FU) (Poudel et al., 2018). The system was further improved by using lauric acid (LA) as gatekeeper of the mesopores, being able to melt at 44°C and thus allowing for a very fine control of drug release. Indeed, the authors reported only *in vitro* studies, which confirmed that 5-FU release was finely controlled by laser irradiation; in fact, without irradiation the release reached only the 10% in 10 h, while upon irradiation it reached the 46% during the same observation time. Moreover, *in vitro* phototoxicity experiments performed on PC3 and DU145 cell lines (Table 1, entry 13), accounted for lower IC_50_ values for 5 FU loaded SGNS-MS-LA NPs (+ irradiation) as respect to free 5-FU and 5-FU loaded SGNS-MS-LA in the absence of laser irradiation (Table 2).

**Table 2:** IC_50_ (μM) of 5-FU and SGNS-MS-LA NPs with and without irradiation on DU145 and PC3 cancer cells.

	**DU145 (IC_**50**_, μM)**	**PC3 (IC_**50**_, μM)**
5-FU	14.71	10.67
SGNS-MS-LA NPs—IRR	30.89	18.76
SGNS-MS-LA NPs + IRR	9.32	7.98

**Figure 11:**
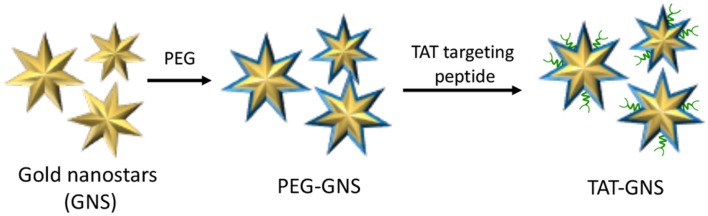
Schematic representation of trans-activating transcriptional (TAT) activator functionalized gold nanostars (GNS).

The main rationale behind nanotechnology-based drug delivery systems is mainly related to the EPR effect that, at certain extent, favors the preferential accumulation of drug-loaded NPs within the tumor tissue through passive targeting (Nakamura et al., 2016). However, EPR effect is not adequate for ensuring to reach the therapeutic concentration at the target site and several surface modification strategies have been developed for augmenting NPs tumor localization (Ferrari, 2010). Nevertheless, the selective accumulation of NPs within the tumor mass as well as their ability to escape the mononuclear phagocyte system still represents a major challenge (Blanco et al., 2015). An alternative approach to enhance the accumulation of NPs within the tumor mass is represented by the use of tumor-tropic cellular vehicles, such as mesenchymal stem cells (MSCs) (Duchi et al., 2013; Näkki et al., 2017). It is well-established that MSCs can be used to effectively transport and deliver either drugs and NPs; moreover, recent studies have demonstrated that drugs and NPs are released from MSCs through micro-vesicles inherently formed within MSCs that would serve as additional bio-carriers (Yeo et al., 2013). In this view, Cheng et al. recently reported the use of MSCs as vehicles to deliver gold nanostars functionalized with the trans-activating transcriptional activator (TAT-GNS) for the PTT treatment of PC (Figure 11) (Huang et al., 2019).

Water-dispersible TAT-GNS of about 100 nm were synthesized by seed-mediated growth approach and further functionalized with PEG chain and TAT targeting peptide to increase GNS cellular interaction. It is worth noting that the anisotropic structure of TAT-GNS strongly contributed to the enhancement of plasmon absorption in the NIR region and subsequent heat formation. The authors thoroughly studied TAT-GNS uptake and cellular localization once treating MSCs, and their results account for very good cells internalization values (determined through ICP mass spectroscopy) up to 44 μg/10^5^ cells at 24 h after incubation with TAT-GNS 160 pM. Moreover, upon TAT-GNS internalization, MSCs retained their vitality and migration ability, which are key features to exert their tumor-tropic properties. In addition, authors were able to demonstrate through TEM analysis, SDS-PAGE and UV-Vis-NIR spectroscopy experiments, that TAT-GSN are released in the culture medium within micro-vesicles, while maintaining their PTT properties. *In vitro* experiments performed on PC3, DU145, and LNCaP cell lines, confirmed the superior antiproliferation activity of MSC loaded with TAT-GNS upon exposure to NIR (800 nm) irradiation. Moreover, MSCs loaded with TAT-GNS maintained “homing” effect and tropic migration ability to improve the GNS intra-tumoral distribution and PTT effect *in vivo* both when administered locally or systematically, providing almost complete tumor regression 16 days after irradiation.

A recent application of PTT for cancer therapy is the near-infrared photoimmunotherapy (NIR-PIT) in which a photothermal agent is conjugated with a molecular antibody (mAb) for the selective targeting of cancer cells (Ogata et al., 2017; Aung et al., 2018; Burley et al., 2018; Kiss et al., 2019). A phase III clinical trial is now recruiting (https://clinicaltrials.gov/ct2/show/record/NCT03769506), involving the intravenous injection of ASP-1929, a cextuximab–IR700 conjugate that selectively binds to EGFR molecules over-expressed on cells membrane of head and neck cancers cells (Kobayashi and Choyke, 2019).

Within this framework, an approach of NIR-PIT for prostate cancer was recently proposed by Kobayashi and collogues, describing the synthesis of an anti-PSMA antibody conjugated with IR700, e.g., anti-PSMA-IR700 (Nagaya et al., 2017). The authors describe the *in vitro* NIR-PIT efficacy of their conjugate, demonstrating that a dose of 3 μg/mL of anti-PSMA-IR700 was able to induce a severe cells' membrane damage exclusively on PSMA-positive PC3pip-luc cells as respect to PSMA-negative PC3flu cells, in a light-dose dependent manner (Table 1, entry 15). In addition, *in vivo* experiments performed on PC3pip-luc xenografted mice, accounted for a strong anti-PSMA-IR700 selective tumor accumulation, and an almost complete tumor reduction upon injection of 100 μg of conjugate followed by two cycles of NIR-PIT. As stated by the authors, these encouraging preliminary results have the potential to be translated to PC patients once established the best settings in terms of light irradiation regimen and stage of the tumor to be treated.

## Vascular PDT for Low-Risk Prostate Cancer

As stated in the introduction, only a fraction of patients affected by PC will develop more significant advanced disease. In turn, the initial stage of the disease is difficult to address and could be prone to over-treatment with invasive surgery and/or radio- and chemotherapy. At this stage, active surveillance is often proposed despite the risk to develop more advanced disease could not be certainly defined and, in order to overcome this limitation, vascular-targeted PDT (VPDT), is becoming a more and more considered therapeutic option (Gheewala et al., 2017; Ritch and Punnen, 2017).

Vascular-targeted PDT is a PDT regimen that preferentially targets abnormal blood vessels through selective delivery of PS molecules to the vasculature (Kraus et al., 2017). Conversely to other therapeutic approaches, such as surgery and chemotherapy, VPDT is less invasive, does not suffer of life-threating drawbacks and, being a physical therapy, is lethal to tumor cells irrespective of their genetic background, as long as they are present within the targeted area (Lee et al., 2017).

**Figure 12:**
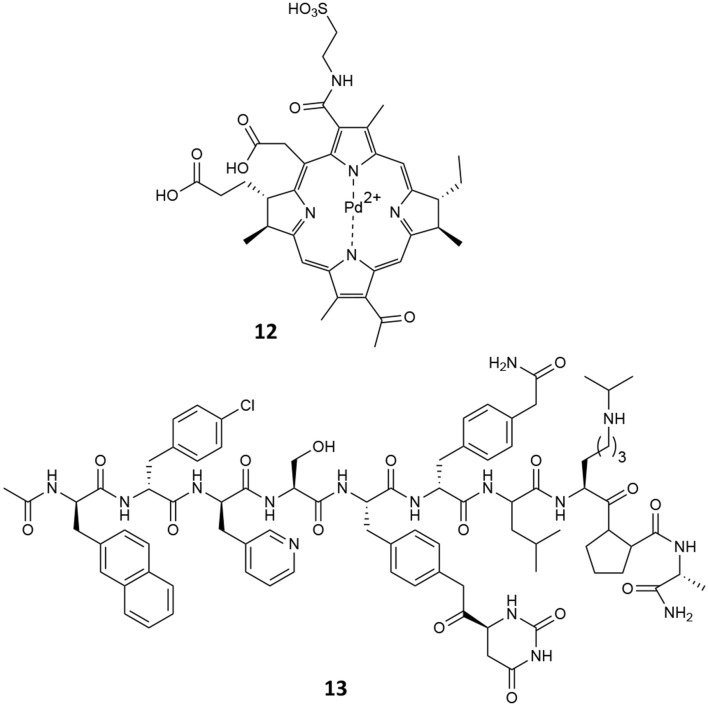
Chemical structures of padeliprofin (**11**) and degarelix (**12**).

In a phase III clinical trial (https://clinicaltrials.gov/ct2/show/NCT01310894), it was demonstrated the VPDT with padeliporfin (**12**, Figure 12) significantly reduced disease progression when compared to active surveillance in patients with low-risk prostate cancer PC (Lebdai et al., 2017). Indeed, low-power NIR light activates padeliporfin, which in turns generates ROS responsible of inducing local vascular occlusion. It is intravenously infused and circulates systemically, while only the targeted area of the prostate is illuminated, inducing irreversible damage to vascular endothelium, quickly followed by vessel occlusion by thrombosis, leading to tumor necrosis (Azzouzi et al., 2017).

However, despite its promising results, tumor recurrence was observed after verteporfin-mediated VPDT and more than 40% patients treated with VPDT modality showed residual tumors in the treated lobes and disease progression (Taneja et al., 2016). In this context, the combination with other treatment modalities has been investigated for improving VPDT outcome. In this view, Coleman et al. recently reported the combination of ADT with degarelix (**13**, Figure 12) and VPDT with padeliporfin in mice bearing LNCaP-AR or VCaP tumors (Kim et al., 2018). In particular, the aim of their study was to identify potential druggable pathways active in PC tumors exposed to VPTD using transcriptome analysis; in fact, the authors established that an acute upregulation of AR pathway activation occurs following VPTD treatment. Thus, they tested the use of degarelix, a long-acting, gonadotropin-releasing hormone antagonist (Broqua et al., 2002), in combination with VPDT, with the aim of inhibiting the AR pro-survival signaling and evaluating the potential co-treatment benefits. To this end, degarelix was administered to nude mice inoculated with either LNCaP-AR or VCaP PC cells, for 3 days prior to VPDT. Animals were then i.v. infused with padeliporfin at the concentration of 9 mg/kg and irradiated with laser light for 10 min (755 nm, 150 mW/cm^2^). Overall their results account for a higher local tumor growth inhibition of DGL/VPDT as compared to single treatments as well as a significant reduction in PSA levels.

## Combined Light-Mediated Therapies

**Figure 13:**
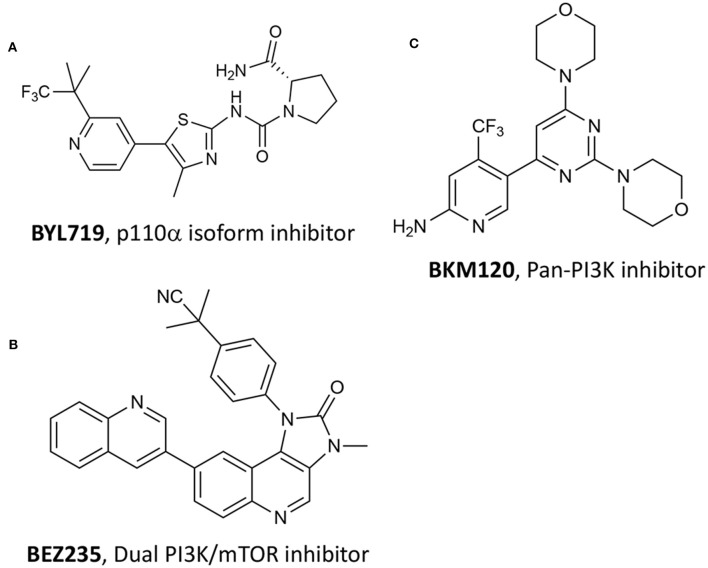
Chemical structures of three P13K inhibitors used in combination with verteporfin VPDT, namely **(A)** BYL719, **(B)** BEZ235 and **(C)** BKM120.

Numerous studies have recently acknowledged the key role of the phosphatidylinositol-4,5-bisphosphate 3-kinase (PI3K)/Ak strain transforming (AKT)/mechanistic target of rapamycin (mTOR) pathway, namely P13K/AKT/mTOR, in PC (Wise et al., 2017; Nevedomskaya et al., 2018). Single-agent treatment with P13K inhibitors and combination with other approaches have been investigated, especially including antiandrogen compounds and chemotherapeutic drugs (Nevedomskaya et al., 2018). Recently, Chen et al. reported the combination of different P13K inhibitors with VPDT (Kraus et al., 2017). The authors hypothesize that by combining P13K/AKT/mTOR pathway inhibition with verteporfin-based VPDT, would have increased the treatment results. In particular, the authors selected three different P13K inhibitors, namely compound BYL719 (Figure 13A) that is selective for the p110α isoform, BKM120 that is a pan-P13K inhibitor (Figure 13B), and BEZ235 that functions as both P13K pan-inhibitor and mTOR kinase inhibitor (Figure 13C) (Maira et al., 2012).

Among the tested P13K inhibitors, BEZ235 resulted the most effective of the three in reducing the viability of SV40 immortalized mouse endothelial cells (SVEC). In addition, by combining P13K inhibitors with verteporfin VPDT at two different verteporfin concentrations, i.e., 200 nM and 400 nM, the dual-target inhibitor BEZ235 was the only one showing a synergistic inhibition of PC3 cells growth at all tested concentrations (Table 1, entry 16). These results prompted the authors to validate their system in pre-clinical animal models (subcutaneous PC3 human prostate tumors in male athymic nude mice) showing that the combined treatment was more effective than VPDT alone, with no tumor recurrence observed 24 days after treatment.

**Figure 14:**
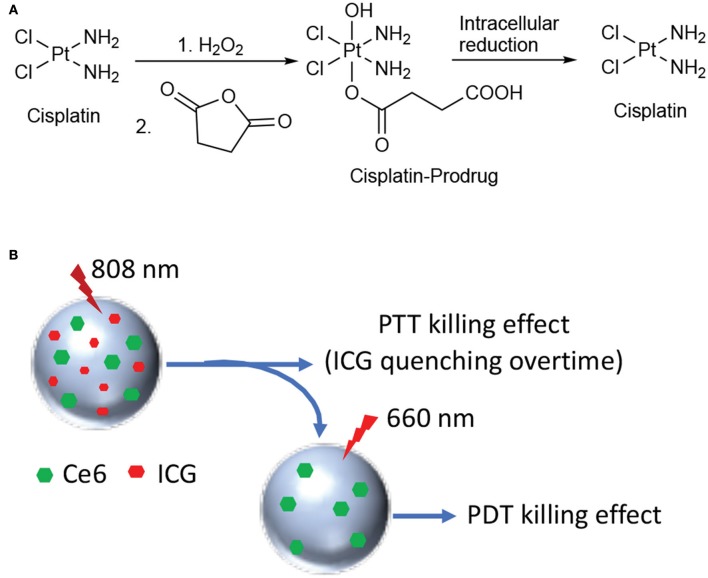
Synthesis of the cisplatin prodrug and mechanism of cisplatin release under reductive conditions **(A)**; schematic representation of HSA NPs loaded with Ce6 and ICG **(B)**.

The combination between hyperthermia therapy and a chemotherapeutic drug was recently explored by Zhang and coworkers for treating PC (Zhang et al., 2018). The authors described the synthesis of novel poly-dopamine NPs covalently functionalized with a cisplatin prodrug (Figure 14A) through a short amino PEG linker. The authors demonstrated both *in vitro* and *in vivo* that cisplatin is released from the particles in reductive intracellular environment, while being able to induce thermal tumor ablation. Pt(IV)-PD-NPs were obtained with a diameter of 74 nm and a Pt(IV) loading of 4.2/w/w and excellent photothermal properties upon 808 nm laser irradiation. *In vitro* cisplatin release experiments were performed in the presence (or not) of 0.1 mM ascorbate, confirming that only in reductive environment the drug is efficiently released in the medium. Moreover, the drug release was further augmented in the presence of increasing doses of light irradiation. *In vitro* experiments were performed on different PC cancer cell lines, showing that in the absence of laser irradiation almost no toxicity was displayed. Similarly, *in vivo* experiments on PC3 xenografts showed that Pt(IV)-PD-NPs had similar cisplatin activity, while upon irradiation tumor was completely eradicated (Table 1, entry 17).

Another example of combination therapy was reported recently by Zhang and coworkers that describe a novel nano-system including human serum albumin (HSA) covalently bound with the photosensitizer Ce6 and simultaneously loaded with the PTT agent indocyanine green (ICG) (Zhang et al., 2018). Dual-loaded NPs were obtained with an average size of 120 nm through desolvation procedure and with excellent encapsulation rates, e.g., 83 and 75% for Ce6 and ICG, respectively. The working mechanism supposed and confirmed by the authors through *in vitro* and *in vivo* experiments is the following: 808 nm laser irradiation only induce excitation of ICG with concomitant production of heat; under these irradiation conditions, Ce6 fluorescence is inhibited and no ROS production takes place. Upon 808 nm irradiation all ICG is degraded and irradiation of the system with 660 nm light restores Ce6 activity, together with the production of cytotoxic ROS (Figure 14B).

The phototherapeutic efficacy of this novel system was evaluated in a nude mouse model bearing PC3 xenografts that were injected with ICG-Ce6-HSA NPs or PBS and illuminated with different NIR laser irradiation 24 h post-injection. After the 808/660 nm laser irradiation, the tumors in the mice treated with dual-loaded NPs significantly decreased and almost disappeared after 15 days of observation (Table 1, entry 18).

## Discussion and Conclusions

Photodynamic (PDT) and photothermal (PTT) therapies are light-induced cytotoxic modalities that gained significant acceptance among the scientific community as they allow a spatial and temporal control of the treatment, while reducing system toxicity (Xiang and Chen, 2019). In particular, PDT employs exogeneous reactive oxygen species and other oxygen-based radicals to kill cancer cells, while PTT exploits the combination of light and special photo-absorbing systems for inducing a local hyper-heating responsible of cells death. Despite both modalities and PDT in particular, have been extensively explored in the last decades as promising strategy for treating tumors, they have not been approved yet as mainstream oncological clinical treatments (Dos Santos et al., 2019), due to some important limitations, such as difficulties in determining the right light dosimetry, the finite and limited penetration of light in deep seated body locations, the lack of sufficient oxygen percentage at the tumor site, the intrinsic toxicity of the used systems, the difficult to selectively damage the tumor tissue, while sparing healthy ones. In this view, problems, cautions, or side effects that can occur during PDT and PTT clinical trials have been recently discussed by other authors, specifically dealing with treatment's settings, outcome and major drawbacks (Triesscheijn et al., 2006; Van Straten et al., 2017; Rastinehad et al., 2019).

Specifically, in the case of PDT light penetration depth and tumor hypoxia constitute major limits to its widespread clinical application. To this end, as lately reported in the literature, several strategies can be undertaken in order to overcome these issues (Mallidi et al., 2016; Zhou et al., 2016). For instance, self-activating strategies exploiting chemo- and bioluminescence can be used, as well as the use of other forms of electromagnetic radiations, such as near-infrared, X-rays or γ-rays can be used for enhancing PDT efficacy. In addition, light source technology has been developed, providing systems with uniform illumination that is an essential condition for improving reproducibility (Stringasci et al., 2017).

Another key point to be considered is tumor hypoxia; this condition is linked to tumor progression and it is an important marker of tumor malignancy as it leads to resistance to several therapeutic approaches, including PDT. Several strategies are in pre-clinical investigation to overwhelm these limitations, including strategies to modify the tumor microenvironment by replenishing oxygen concentration, as well as the use of oxygen carriers or self-oxygen generating systems, e.g., red blood cells, perfluorocarbon NPs, etc. (Dang et al., 2017; Luo et al., 2018; Banerjee, 2019).

In this paper we aimed at reviewing the last 5-years literature on the use of light-mediated therapies for the treatment of prostate cancer; in particular we wanted to provide an overview of this topic based on both the chemical and the biological point of view. Overall, the articles discussed herein confirm that chemical synthesis and material chemistry are key-tools for the production of multi-modal conjugates as well as nano-platforms able to carry multiples features, such as different therapeutic agents, including PS for light-mediated treatment, as well as specific prostate cancer targeting antennas for improving the treatment selectivity, and imaging probes.

However, the analysis of *in vitro* and *in vivo* data herein reported, show that in many cases PDT settings lack consistency and rigorous approach, which weaken its reproducibility and uniformity even at the preclinical level. Future directions on the use of PDT for cancer treatment including PC, should consider the standardization of light sources and light dosimetry technologies; more studies should focus on toxicology especially when including complex nanomaterials, since this aspect is of paramount importance for devising a future clinical translation.

Indeed, in spite both PDT and PTT lack of systemic toxicity exerting mainly a local effect, and because they can be easily used in combination with other treatment modalities, due to their unique mechanism of action, only very few PS have been translated into clinical application. Currently nearly 50 clinical trials are ongoing using already approved PS for the treatment of superficial tumors (Dos Santos et al., 2019). Specifically, Phase II and III clinical trials on the vascular PDT treatment of prostate cancer using WST11, brand name TOOKAD®, have shown promising results, and additional trails are ongoing for assessing its safety (https://clinicaltrials.gov/ct2/show/NCT03315754, https://clinicaltrials.gov/ct2/show/NCT03849365).

In conclusion, the current analysis of the state-of-the-art shows that PDT and PTT represent promising and effective approaches for the combined treatment of PC especially when the disease is still confined to the prostate region. However, more research is ongoing to bring light-induced therapies at the forefront of PC treatment.

## Author Contributions

GV conceived, organized, and drafted the paper. CF, AD, EM, and CM contributed to paper writing, figures drawing, and overall revision.

### Conflict of Interest

AD was CEO and Shareholder in the company Innovamol Consulting Srl. The remaining authors declare that the research was conducted in the absence of any commercial or financial relationships that could be construed as a potential conflict of interest.
